# Bis(9-amino­acridinium) bis­(pyridine-2,6-dicarboxyl­ato-κ^3^ *O* ^2^,*N*,*O* ^6^)nickelate(II) trihydrate

**DOI:** 10.1107/S1600536810016776

**Published:** 2010-05-15

**Authors:** Zohreh Derikvand, Marilyn M. Olmstead

**Affiliations:** a Department of Chemistry, Faculty of Sciences, Islamic Azad University, Khorramabad Branch, Khorramabad, Iran; b Department of Chemistry, University of California, One Shields Avenue, Davis, CA 95616-5292, USA

## Abstract

The title compound, (C_13_H_11_N_2_)_2_[Ni(C_7_H_3_NO_4_)_2_]·3H_2_O, consists of a mononuclear anionic complex, two 9-amino­acridinium cations and three uncoordinated water mol­ecules. Two pyridine-2,6-dicarboxyl­ate (pydc) ligands are bound to the Ni^II^ ion, giving an NiN_2_O_4_ bonded set. The coordination geometry around the Ni^II^ atom is distorted octa­hedral. There are two types of robust O—H⋯O hydrogen-bond synthons, namely *R*_6_^6^(24) and *R*_2_^4^(8), which link the complex anions and water mol­ecules to each other. N—H⋯O hydrogen bonds connect the stacks of anions and cations in the structure. Other inter­molecular inter­actions, including weak C—H⋯O hydrogen bonds, π–π [shortest centroid–centroid distance = 3.336 (7) Å] and C—O⋯π [O⋯centroid distance = 3.562 (10) Å] inter­actions, connect the various components.

## Related literature

For related structures containing [Ni(pydc)_2_]^2−^ species, see: Aghabozorg *et al.* (2008 ▶, 2009 ▶); Attar Gharamaleki *et al.* (2009 ▶); Cui *et al.* (2009 ▶); Hadadzadeh *et al.* (2010 ▶); Safaei-Ghomi *et al.* (2009 ▶).
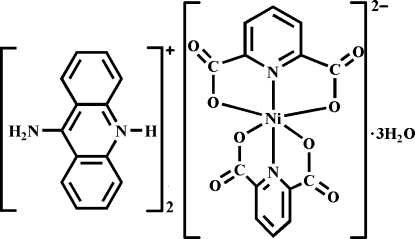


## Experimental

### 

#### Crystal data

(C_13_H_11_N_2_)_2_[Ni(C_7_H_3_NO_4_)_2_]·3H_2_O
*M* *_r_* = 833.44
Triclinic, 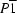

*a* = 10.7939 (10) Å
*b* = 13.3335 (12) Å
*c* = 13.9370 (13) Å
α = 102.288 (2)°
β = 103.609 (2)°
γ = 105.482 (2)°
*V* = 1795.6 (3) Å^3^
*Z* = 2
Mo *K*α radiation
μ = 0.62 mm^−1^
*T* = 90 K
0.36 × 0.24 × 0.20 mm

#### Data collection

Bruker APEXII CCD diffractometer
Absorption correction: multi-scan (*SADABS*; Sheldrick, 1996 ▶) *T*_min_ = 0.809, *T*_max_ = 0.887
29993 measured reflections
11816 independent reflections
10951 reflections with *I* > 2σ(*I*)
*R*_int_ = 0.015

#### Refinement

*R*[*F* ^2^ > 2σ(*F* ^2^)] = 0.027
*wR*(*F* ^2^) = 0.076
*S* = 1.04
11816 reflections
659 parameters
All H-atom parameters refined
Δρ_max_ = 0.48 e Å^−3^
Δρ_min_ = −0.34 e Å^−3^

### 

Data collection: *APEX2* (Bruker, 2007 ▶); cell refinement: *SAINT* (Bruker, 2007 ▶); data reduction: *SAINT*; program(s) used to solve structure: *SHELXS97* (Sheldrick, 2008 ▶); program(s) used to refine structure: *SHELXL97* (Sheldrick, 2008 ▶); molecular graphics: *SHELXTL* (Sheldrick, 2008 ▶) and *Mercury* (Macrae *et al.*, 2006 ▶); software used to prepare material for publication: *SHELXL97*.

## Supplementary Material

Crystal structure: contains datablocks I, global. DOI: 10.1107/S1600536810016776/hy2305sup1.cif

Structure factors: contains datablocks I. DOI: 10.1107/S1600536810016776/hy2305Isup2.hkl

Additional supplementary materials:  crystallographic information; 3D view; checkCIF report

## Figures and Tables

**Table 1:** Hydrogen-bond geometry (Å, °)

*D*—H⋯*A*	*D*—H	H⋯*A*	*D*⋯*A*	*D*—H⋯*A*
N3—H3*A*⋯O2*W*^i^	0.873 (18)	2.053 (18)	2.8793 (12)	157.6 (16)
N3—H3*B*⋯O7^ii^	0.878 (17)	2.109 (17)	2.9337 (11)	156.1 (15)
N4—H4*A*⋯O6	0.884 (17)	1.838 (17)	2.7214 (11)	178.1 (16)
N5—H5*A*⋯O3^iii^	0.894 (17)	1.925 (17)	2.7945 (11)	163.9 (16)
N5—H5*B*⋯O5	0.889 (17)	2.046 (17)	2.9096 (11)	163.6 (15)
N6—H6*A*⋯O3*W*	0.836 (18)	1.863 (18)	2.6903 (11)	170.3 (18)
O1*W*—H1*A*⋯O7	0.83 (2)	1.99 (2)	2.8138 (11)	171 (2)
O1*W*—H1*B*⋯O7^iv^	0.861 (19)	1.955 (19)	2.8161 (11)	178.7 (17)
O2*W*—H2*A*⋯O8^v^	0.813 (19)	2.067 (19)	2.8717 (11)	170.1 (17)
O2*W*—H2*B*⋯O2	0.79 (2)	2.02 (2)	2.8033 (11)	177 (2)
O3*W*—H3*C*⋯O3^vi^	0.79 (2)	1.97 (2)	2.7595 (11)	169.8 (19)
O3*W*—H3*D*⋯O1*W*^vii^	0.856 (19)	1.879 (19)	2.7328 (11)	175.4 (17)
C30—H30⋯O3^iii^	0.931 (16)	2.437 (16)	3.3386 (12)	163 (14)

Symmetry codes: (i) 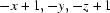; (ii) 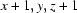; (iii) 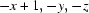; (iv) 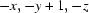; (v) 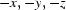; (vi) 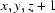; (vii) 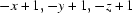.

**Fig. 1.**
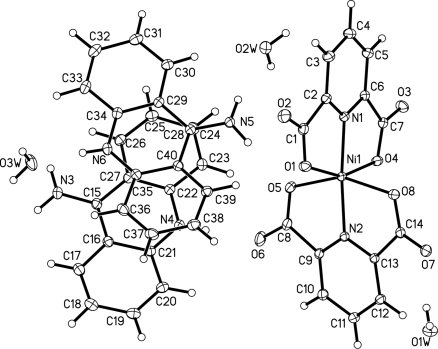
Molecular structure of the title compound. Displacement ellipsoids are shown at the 50% probability level.

**Fig. 2.**
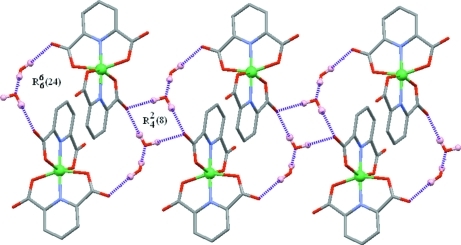
A view of the two types of robust hydrogen bond synthons, R66(24) and R24(8), which link the complex anions and water molecules to each other.

**Fig. 3.**
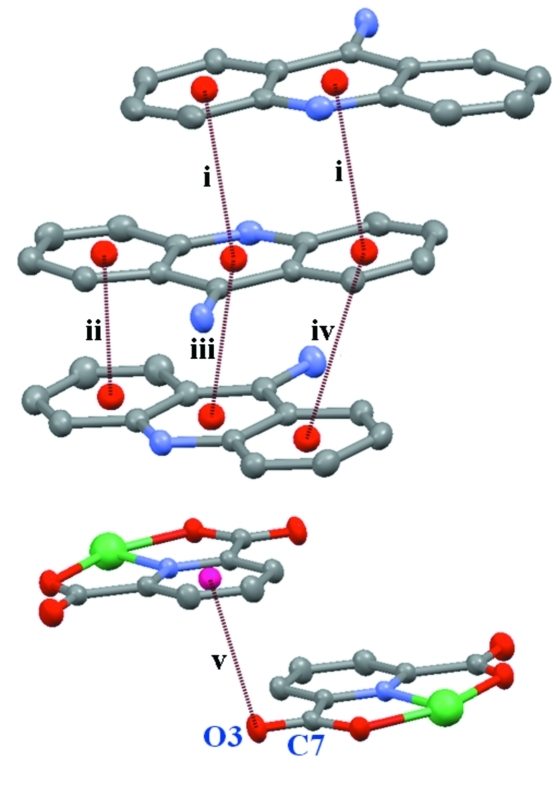
A view of the extensive π–π stacking interactions (dashed lines) between aromatic rings of 9-aminoacridinium ions and the C—O···π interaction (dashed line) between C7—O3 and the centroid of the pyridyl ring of a neighboring pydc ligand. [Distances: (i) 3.336; (ii) 3.834; (iii) 3.429; (iv) 3.621; (v) 3.562 Å.]

**Fig. 4.**
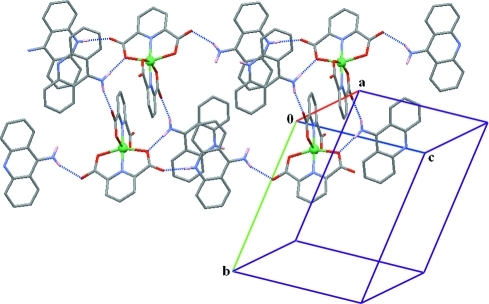
A view showing how the cations and anions are connected by N—H···O hydrogen bonds (dashed lines).

## supplementary crystallographic information

### Comment

9-Aminoacridine is a highly fluorescent dye clinically used as a topical antiseptic and experimentally as a mutagen, an intracellular pH indicator and as a MALDI matrix. Acridine and related derivatives bind to DNA and RNA due to their abilities to intercalate. Many complexes containing pyridine-2,6-dicarboxylate (pydc), Ni^II^ ions and various bases have been reported (Aghabozorg *et al.,* 2008, 2009; Attar Gharamaleki *et al.,* 2009; Cui *et al.*, 2009; Hadadzadeh *et al.*, 2010; Safaei-Ghomi *et al.,* 2009).

The asymmetric unit of the title compound consists of one [Ni(pydc)_2_]^2-^ anion, two 9-aminoacridinuum cations and three uncoordinated water molecules (Fig. 1). In the synthesis, two carboxylic acid protons are transferred to the endocyclic N atoms of 9-aminoacridines. Two pydc ligands are bound to the metal ion to give a NiN_2_O_4_ bonded set, using all the coordination sites of the pydc ligand. The resulting coordination polyhedron can be described as distorted octahedral. In the anionic complex, the Ni—N bond distances [1.9648 (8) and 1.9760 (8) Å] are shorter than the Ni—O bond distances [2.1003 (7), 2.1120 (7), 2.1360 (7) and 2.1776 (7) Å]. It is worth pointing out that there are two types of robust hydrogen bond synthons, namely R^6^_6_(24) and R^2^_4_(8), which link the complex anions and water molecules to each other, as shown in Fig. 2. The dihedral angle between two coordinated pydc ligands is 85.14 (3)°, which shows that two ligands are almost perpendicular to each other. A feature of the title compound is the presence of π–π and C—O···π interactions. The shortest π–π distance is 3.336 (7) Å and C—O···π distances is 3.562 (10) Å (Fig. 3). Another feature in this crystal structure is intermolecular O—H···O, N—H···O and C—H···O hydrogen bonds with D···A distances ranging from 2.6903 (11) to 3.3386 (12)Å (Table 1).

The packing consists of distinctive stacks of cations and anions that propagate along the *a* direction. These stacks are connected to each other by N—H···O hydrogen bonds, as shown in Fig. 4. This crystal structure is comparable to (H_3_O)^+^(creatH)^+^[Ni(pydc)_2_].3H_2_O (creat = creatinine) (Attar Gharamaleki *et al.*, 2009).

### Experimental

An aqueous solution of Ni(NO_3_)_2_.6H_2_O (145 mg, 0.5 mmol) in distilled water (5 ml) was added to methanolic solution of pyridine-2,6-dicarboxylic acid (167 mg, 1 mmol) in water (20 ml) and 9-aminoacridine (194 mg, 1 mmol) in methanol (5 ml) under stirring at 80°C in a 1:2:2 molar ratio. The green colored precipitated product was obtained. The precipitation was dissolved in solution of H_2_O/DMSO in a volume ratio of 1:2 (5/10 ml). Green block crystals, suitable for X-ray characterization, were obtained after 4 d at room temperature.

### Refinement

All H atoms were located in difference Fourier maps and refined isotropically.

### Crystal data

**Table:**

(C_13_H_11_N_2_)_2_[Ni(C_7_H_3_NO_4_)_2_]·3H_2_O	*Z* = 2
*M**_r_* = 833.44	*F*(000) = 864
Triclinic, *P*1	*D*_x_ = 1.542 Mg m^−^^3^
Hall symbol: -P 1	Mo *K*α radiation, λ = 0.71073 Å
*a* = 10.7939 (10) Å	Cell parameters from 9659 reflections
*b* = 13.3335 (12) Å	θ = 2.9–31.5°
*c* = 13.9370 (13) Å	µ = 0.62 mm^−^^1^
α = 102.288 (2)°	*T* = 90 K
β = 103.609 (2)°	Block, green
γ = 105.482 (2)°	0.36 × 0.24 × 0.20 mm
*V* = 1795.6 (3) Å^3^	

### Data collection

**Table:**

Bruker APEXII CCD diffractometer	11816 independent reflections
Radiation source: fine-focus sealed tube	10951 reflections with *I* > 2σ(*I*)
graphite	*R*_int_ = 0.015
Detector resolution: 0.83 pixels mm^-1^	θ_max_ = 31.5°, θ_min_ = 2.8°
φ and ω scans	*h* = −15→15
Absorption correction: multi-scan (*SADABS*; Sheldrick, 1996)	*k* = −19→19
*T*_min_ = 0.809, *T*_max_ = 0.887	*l* = −20→20
29993 measured reflections	

### Refinement

**Table:**

Refinement on *F*^2^	Primary atom site location: structure-invariant direct methods
Least-squares matrix: full	Secondary atom site location: difference Fourier map
*R*[*F*^2^ > 2σ(*F*^2^)] = 0.027	Hydrogen site location: difference Fourier map
*wR*(*F*^2^) = 0.076	All H-atom parameters refined
*S* = 1.04	*w* = 1/[σ^2^(*F*_o_^2^) + (0.0406*P*)^2^ + 0.6602*P*] where *P* = (*F*_o_^2^ + 2*F*_c_^2^)/3
11816 reflections	(Δ/σ)_max_ = 0.008
659 parameters	Δρ_max_ = 0.48 e Å^−^^3^
0 restraints	Δρ_min_ = −0.34 e Å^−^^3^

### Fractional atomic coordinates and isotropic or equivalent isotropic displacement parameters (Å^2^)

**Table:**

	*x*	*y*	*z*	*U*_iso_*/*U*_eq_	
Ni1	0.359601 (12)	0.243203 (9)	0.083985 (9)	0.01101 (3)	
O1	0.24161 (7)	0.20633 (6)	0.18019 (5)	0.01602 (13)	
O2	0.09729 (8)	0.06546 (6)	0.20554 (6)	0.01894 (14)	
O3	0.47436 (8)	0.07283 (6)	−0.14184 (5)	0.01698 (13)	
O4	0.45471 (7)	0.21101 (5)	−0.02971 (5)	0.01462 (12)	
O5	0.54671 (7)	0.27912 (6)	0.20078 (5)	0.01500 (12)	
O6	0.73195 (8)	0.42266 (7)	0.30005 (6)	0.02066 (15)	
O7	0.14490 (7)	0.42729 (6)	−0.04067 (5)	0.01533 (13)	
O8	0.20247 (7)	0.28295 (5)	−0.01471 (5)	0.01506 (12)	
N1	0.28850 (8)	0.08335 (6)	0.03374 (6)	0.01215 (13)	
N2	0.43332 (8)	0.40357 (6)	0.11994 (6)	0.01076 (13)	
C1	0.17544 (10)	0.10551 (8)	0.16048 (7)	0.01420 (16)	
C2	0.20048 (9)	0.02963 (7)	0.07382 (7)	0.01305 (15)	
C3	0.14477 (10)	−0.08340 (8)	0.03748 (8)	0.01671 (17)	
H3	0.0808 (16)	−0.1233 (13)	0.0667 (12)	0.026 (4)*	
C4	0.18596 (11)	−0.13846 (8)	−0.03952 (8)	0.01748 (17)	
H4	0.1504 (15)	−0.2174 (13)	−0.0647 (12)	0.023 (4)*	
C5	0.27985 (10)	−0.08048 (8)	−0.07890 (7)	0.01533 (16)	
H5	0.3120 (16)	−0.1156 (13)	−0.1310 (12)	0.024 (4)*	
C6	0.32823 (9)	0.03235 (7)	−0.04062 (7)	0.01215 (15)	
C7	0.42722 (9)	0.11096 (7)	−0.07411 (7)	0.01279 (15)	
C8	0.61936 (10)	0.37940 (8)	0.23293 (7)	0.01360 (15)	
C9	0.55891 (9)	0.45375 (7)	0.18393 (7)	0.01160 (15)	
C10	0.62363 (9)	0.56455 (8)	0.20244 (7)	0.01367 (15)	
H10	0.7148 (16)	0.5980 (13)	0.2491 (12)	0.025 (4)*	
C11	0.55385 (10)	0.62207 (7)	0.15184 (7)	0.01395 (16)	
H11	0.5949 (15)	0.6953 (12)	0.1617 (11)	0.019 (3)*	
C12	0.42193 (9)	0.56827 (7)	0.08503 (7)	0.01255 (15)	
H12	0.3741 (15)	0.6076 (12)	0.0478 (11)	0.019 (3)*	
C13	0.36452 (9)	0.45752 (7)	0.07089 (7)	0.01066 (14)	
C14	0.22569 (9)	0.38391 (7)	−0.00075 (7)	0.01211 (15)	
N3	1.01535 (9)	0.30800 (7)	0.73932 (6)	0.01601 (15)	
H3A	1.0262 (18)	0.2447 (15)	0.7348 (13)	0.035 (4)*	
H3B	1.0349 (16)	0.3518 (14)	0.8016 (13)	0.028 (4)*	
N4	0.85905 (8)	0.38995 (6)	0.47807 (6)	0.01216 (13)	
H4A	0.8176 (17)	0.4019 (13)	0.4211 (13)	0.029 (4)*	
C15	0.96750 (9)	0.33591 (7)	0.65564 (7)	0.01238 (15)	
C16	0.92971 (9)	0.43231 (7)	0.66353 (7)	0.01277 (15)	
C17	0.94175 (10)	0.50324 (8)	0.75957 (7)	0.01699 (17)	
H17	0.9776 (15)	0.4885 (12)	0.8207 (12)	0.022 (4)*	
C18	0.89975 (11)	0.59228 (8)	0.76344 (8)	0.01865 (18)	
H18	0.9081 (15)	0.6410 (12)	0.8279 (12)	0.023 (4)*	
C19	0.84486 (10)	0.61508 (8)	0.67144 (8)	0.01701 (17)	
H19	0.8169 (17)	0.6750 (13)	0.6743 (13)	0.029 (4)*	
C20	0.83206 (10)	0.54841 (8)	0.57722 (8)	0.01467 (16)	
H20	0.7949 (15)	0.5620 (12)	0.5123 (12)	0.024 (4)*	
C21	0.87392 (9)	0.45595 (7)	0.57219 (7)	0.01210 (15)	
C22	0.89545 (9)	0.29898 (7)	0.46724 (7)	0.01138 (14)	
C23	0.87734 (9)	0.23509 (8)	0.36660 (7)	0.01369 (15)	
H23	0.8404 (15)	0.2582 (12)	0.3080 (11)	0.019 (3)*	
C24	0.91252 (10)	0.14290 (8)	0.35392 (7)	0.01527 (16)	
H24	0.9000 (16)	0.1003 (13)	0.2858 (12)	0.027 (4)*	
C25	0.96692 (9)	0.11129 (8)	0.44075 (8)	0.01504 (16)	
H25	0.9882 (15)	0.0445 (12)	0.4304 (11)	0.021 (4)*	
C26	0.98706 (9)	0.17326 (8)	0.53888 (7)	0.01362 (15)	
H26	1.0261 (15)	0.1520 (12)	0.5973 (11)	0.020 (3)*	
C27	0.95095 (9)	0.26932 (7)	0.55488 (7)	0.01163 (15)	
N5	0.54540 (8)	0.10654 (7)	0.29830 (6)	0.01346 (14)	
H5B	0.5307 (17)	0.1587 (14)	0.2716 (12)	0.028 (4)*	
H5A	0.5530 (17)	0.0488 (14)	0.2569 (13)	0.032 (4)*	
N6	0.66618 (8)	0.17508 (7)	0.61591 (6)	0.01321 (14)	
H6A	0.6815 (18)	0.1842 (14)	0.6793 (14)	0.035 (4)*	
C28	0.58088 (9)	0.12538 (7)	0.39986 (7)	0.01094 (14)	
C29	0.63617 (9)	0.05563 (7)	0.44931 (7)	0.01157 (14)	
C30	0.65177 (9)	−0.04016 (7)	0.39368 (7)	0.01397 (15)	
H30	0.6252 (15)	−0.0608 (12)	0.3218 (12)	0.020 (3)*	
C31	0.70392 (10)	−0.10461 (8)	0.44482 (8)	0.01636 (17)	
H31	0.7130 (15)	−0.1690 (13)	0.4074 (12)	0.021 (3)*	
C32	0.74404 (9)	−0.07552 (8)	0.55373 (8)	0.01687 (17)	
H32	0.7815 (15)	−0.1210 (12)	0.5896 (12)	0.023 (4)*	
C33	0.73293 (9)	0.01756 (8)	0.61017 (8)	0.01544 (16)	
H33	0.7632 (16)	0.0396 (13)	0.6863 (12)	0.027 (4)*	
C34	0.67789 (9)	0.08377 (7)	0.55852 (7)	0.01229 (15)	
C35	0.60723 (9)	0.23954 (7)	0.57198 (7)	0.01228 (15)	
C36	0.58907 (10)	0.32831 (8)	0.63673 (7)	0.01603 (17)	
H36	0.6224 (16)	0.3430 (13)	0.7095 (13)	0.027 (4)*	
C37	0.52273 (11)	0.38982 (8)	0.59321 (8)	0.01782 (17)	
H37	0.5095 (16)	0.4525 (13)	0.6367 (12)	0.026 (4)*	
C38	0.47143 (10)	0.36511 (8)	0.48487 (8)	0.01641 (17)	
H38	0.4195 (15)	0.4061 (12)	0.4546 (11)	0.021 (4)*	
C39	0.49140 (9)	0.28085 (7)	0.42143 (7)	0.01346 (15)	
H39	0.4518 (14)	0.2641 (12)	0.3479 (11)	0.017 (3)*	
C40	0.56196 (9)	0.21681 (7)	0.46364 (7)	0.01130 (14)	
O1W	0.11651 (8)	0.63289 (6)	0.02097 (6)	0.01961 (14)	
H1A	0.132 (2)	0.5745 (16)	0.0089 (15)	0.045 (5)*	
H1B	0.0366 (19)	0.6155 (14)	0.0272 (13)	0.033 (4)*	
O2W	−0.08266 (9)	−0.12483 (7)	0.21495 (6)	0.02072 (15)	
H2A	−0.1191 (18)	−0.1637 (15)	0.1557 (14)	0.033 (4)*	
H2B	−0.031 (2)	−0.0729 (16)	0.2114 (15)	0.041 (5)*	
O3W	0.68638 (9)	0.20630 (7)	0.81693 (6)	0.02351 (16)	
H3C	0.628 (2)	0.1734 (16)	0.8358 (14)	0.039 (5)*	
H3D	0.7472 (19)	0.2544 (15)	0.8698 (14)	0.035 (4)*	

### Atomic displacement parameters (Å^2^)

**Table:**

	*U*^11^	*U*^22^	*U*^33^	*U*^12^	*U*^13^	*U*^23^
Ni1	0.01530 (6)	0.00874 (5)	0.01001 (5)	0.00488 (4)	0.00442 (4)	0.00317 (4)
O1	0.0215 (3)	0.0127 (3)	0.0155 (3)	0.0059 (3)	0.0089 (3)	0.0036 (2)
O2	0.0230 (3)	0.0193 (3)	0.0185 (3)	0.0069 (3)	0.0120 (3)	0.0076 (3)
O3	0.0217 (3)	0.0153 (3)	0.0143 (3)	0.0052 (3)	0.0095 (3)	0.0019 (2)
O4	0.0214 (3)	0.0106 (3)	0.0139 (3)	0.0057 (2)	0.0081 (2)	0.0042 (2)
O5	0.0196 (3)	0.0138 (3)	0.0142 (3)	0.0080 (2)	0.0048 (2)	0.0067 (2)
O6	0.0178 (3)	0.0236 (4)	0.0188 (3)	0.0065 (3)	−0.0008 (3)	0.0105 (3)
O7	0.0145 (3)	0.0158 (3)	0.0152 (3)	0.0072 (2)	0.0014 (2)	0.0044 (2)
O8	0.0166 (3)	0.0112 (3)	0.0148 (3)	0.0038 (2)	0.0018 (2)	0.0030 (2)
N1	0.0163 (3)	0.0105 (3)	0.0107 (3)	0.0052 (3)	0.0048 (3)	0.0036 (3)
N2	0.0133 (3)	0.0105 (3)	0.0097 (3)	0.0054 (3)	0.0039 (3)	0.0033 (2)
C1	0.0173 (4)	0.0153 (4)	0.0121 (4)	0.0071 (3)	0.0054 (3)	0.0050 (3)
C2	0.0164 (4)	0.0120 (4)	0.0121 (4)	0.0051 (3)	0.0053 (3)	0.0050 (3)
C3	0.0208 (4)	0.0128 (4)	0.0166 (4)	0.0036 (3)	0.0074 (3)	0.0052 (3)
C4	0.0230 (5)	0.0107 (4)	0.0166 (4)	0.0031 (3)	0.0063 (3)	0.0029 (3)
C5	0.0212 (4)	0.0113 (4)	0.0131 (4)	0.0053 (3)	0.0055 (3)	0.0026 (3)
C6	0.0162 (4)	0.0107 (3)	0.0101 (3)	0.0051 (3)	0.0042 (3)	0.0033 (3)
C7	0.0165 (4)	0.0121 (4)	0.0105 (3)	0.0052 (3)	0.0044 (3)	0.0041 (3)
C8	0.0162 (4)	0.0161 (4)	0.0122 (4)	0.0082 (3)	0.0054 (3)	0.0071 (3)
C9	0.0138 (4)	0.0126 (4)	0.0101 (3)	0.0058 (3)	0.0041 (3)	0.0046 (3)
C10	0.0135 (4)	0.0137 (4)	0.0127 (4)	0.0035 (3)	0.0028 (3)	0.0043 (3)
C11	0.0161 (4)	0.0113 (4)	0.0147 (4)	0.0040 (3)	0.0049 (3)	0.0048 (3)
C12	0.0151 (4)	0.0116 (4)	0.0130 (4)	0.0062 (3)	0.0050 (3)	0.0048 (3)
C13	0.0124 (4)	0.0107 (3)	0.0097 (3)	0.0051 (3)	0.0036 (3)	0.0032 (3)
C14	0.0132 (4)	0.0129 (4)	0.0101 (3)	0.0045 (3)	0.0034 (3)	0.0033 (3)
N3	0.0186 (4)	0.0169 (4)	0.0116 (3)	0.0068 (3)	0.0016 (3)	0.0047 (3)
N4	0.0133 (3)	0.0124 (3)	0.0116 (3)	0.0051 (3)	0.0034 (3)	0.0045 (3)
C15	0.0101 (3)	0.0132 (4)	0.0124 (4)	0.0028 (3)	0.0022 (3)	0.0039 (3)
C16	0.0118 (4)	0.0128 (4)	0.0125 (4)	0.0035 (3)	0.0027 (3)	0.0031 (3)
C17	0.0185 (4)	0.0167 (4)	0.0134 (4)	0.0055 (3)	0.0031 (3)	0.0022 (3)
C18	0.0203 (4)	0.0162 (4)	0.0168 (4)	0.0055 (3)	0.0052 (3)	0.0008 (3)
C19	0.0168 (4)	0.0133 (4)	0.0206 (4)	0.0056 (3)	0.0064 (3)	0.0029 (3)
C20	0.0142 (4)	0.0130 (4)	0.0178 (4)	0.0054 (3)	0.0050 (3)	0.0054 (3)
C21	0.0107 (3)	0.0116 (4)	0.0135 (4)	0.0032 (3)	0.0038 (3)	0.0036 (3)
C22	0.0101 (3)	0.0120 (3)	0.0125 (4)	0.0036 (3)	0.0038 (3)	0.0044 (3)
C23	0.0142 (4)	0.0154 (4)	0.0121 (4)	0.0054 (3)	0.0043 (3)	0.0046 (3)
C24	0.0156 (4)	0.0162 (4)	0.0149 (4)	0.0064 (3)	0.0062 (3)	0.0034 (3)
C25	0.0136 (4)	0.0147 (4)	0.0186 (4)	0.0066 (3)	0.0059 (3)	0.0049 (3)
C26	0.0119 (4)	0.0143 (4)	0.0160 (4)	0.0055 (3)	0.0042 (3)	0.0060 (3)
C27	0.0100 (3)	0.0124 (4)	0.0124 (4)	0.0036 (3)	0.0030 (3)	0.0043 (3)
N5	0.0185 (4)	0.0127 (3)	0.0107 (3)	0.0076 (3)	0.0045 (3)	0.0037 (3)
N6	0.0130 (3)	0.0163 (3)	0.0101 (3)	0.0042 (3)	0.0037 (3)	0.0043 (3)
C28	0.0096 (3)	0.0113 (3)	0.0118 (3)	0.0029 (3)	0.0037 (3)	0.0035 (3)
C29	0.0105 (3)	0.0121 (4)	0.0128 (4)	0.0038 (3)	0.0038 (3)	0.0049 (3)
C30	0.0131 (4)	0.0127 (4)	0.0165 (4)	0.0047 (3)	0.0045 (3)	0.0047 (3)
C31	0.0142 (4)	0.0140 (4)	0.0228 (4)	0.0058 (3)	0.0060 (3)	0.0075 (3)
C32	0.0121 (4)	0.0184 (4)	0.0235 (5)	0.0054 (3)	0.0056 (3)	0.0126 (4)
C33	0.0120 (4)	0.0197 (4)	0.0167 (4)	0.0048 (3)	0.0042 (3)	0.0105 (3)
C34	0.0095 (3)	0.0145 (4)	0.0130 (4)	0.0027 (3)	0.0037 (3)	0.0057 (3)
C35	0.0112 (3)	0.0132 (4)	0.0117 (4)	0.0025 (3)	0.0045 (3)	0.0032 (3)
C36	0.0180 (4)	0.0159 (4)	0.0127 (4)	0.0037 (3)	0.0067 (3)	0.0016 (3)
C37	0.0224 (4)	0.0149 (4)	0.0179 (4)	0.0068 (3)	0.0107 (4)	0.0026 (3)
C38	0.0186 (4)	0.0141 (4)	0.0191 (4)	0.0072 (3)	0.0083 (3)	0.0052 (3)
C39	0.0145 (4)	0.0131 (4)	0.0141 (4)	0.0054 (3)	0.0054 (3)	0.0046 (3)
C40	0.0112 (3)	0.0115 (3)	0.0113 (3)	0.0036 (3)	0.0042 (3)	0.0030 (3)
O1W	0.0175 (3)	0.0149 (3)	0.0243 (4)	0.0041 (3)	0.0058 (3)	0.0040 (3)
O2W	0.0258 (4)	0.0174 (3)	0.0157 (3)	0.0039 (3)	0.0037 (3)	0.0055 (3)
O3W	0.0238 (4)	0.0272 (4)	0.0120 (3)	−0.0023 (3)	0.0057 (3)	0.0041 (3)

### Geometric parameters (Å, °)

**Table:**

Ni1—N1	1.9648 (8)	C18—H18	0.959 (15)
Ni1—N2	1.9760 (8)	C19—C20	1.3732 (14)
Ni1—O1	2.1003 (7)	C19—H19	0.923 (17)
Ni1—O4	2.1120 (7)	C20—C21	1.4158 (12)
Ni1—O5	2.1360 (7)	C20—H20	0.974 (15)
Ni1—O8	2.1776 (7)	C22—C27	1.4124 (12)
O1—C1	1.2770 (12)	C22—C23	1.4158 (12)
O2—C1	1.2420 (12)	C23—C24	1.3692 (13)
O3—C7	1.2523 (11)	C23—H23	0.969 (15)
O4—C7	1.2655 (11)	C24—C25	1.4148 (13)
O5—C8	1.2741 (12)	C24—H24	0.955 (16)
O6—C8	1.2434 (12)	C25—C26	1.3725 (13)
O7—C14	1.2580 (11)	C25—H25	0.968 (15)
O8—C14	1.2642 (11)	C26—C27	1.4240 (12)
N1—C2	1.3359 (12)	C26—H26	0.960 (15)
N1—C6	1.3361 (11)	N5—C28	1.3249 (11)
N2—C9	1.3318 (12)	N5—H5B	0.889 (17)
N2—C13	1.3385 (11)	N5—H5A	0.894 (17)
C1—C2	1.5280 (13)	N6—C35	1.3606 (12)
C2—C3	1.3919 (13)	N6—C34	1.3613 (12)
C3—C4	1.3970 (14)	N6—H6A	0.836 (18)
C3—H3	0.977 (16)	C28—C29	1.4393 (12)
C4—C5	1.3963 (14)	C28—C40	1.4405 (12)
C4—H4	0.971 (16)	C29—C34	1.4157 (12)
C5—C6	1.3888 (12)	C29—C30	1.4197 (12)
C5—H5	0.963 (16)	C30—C31	1.3760 (13)
C6—C7	1.5176 (13)	C30—H30	0.930 (15)
C8—C9	1.5162 (12)	C31—C32	1.4116 (14)
C9—C10	1.3925 (13)	C31—H31	0.947 (15)
C10—C11	1.3934 (13)	C32—C33	1.3717 (14)
C10—H10	0.965 (16)	C32—H32	0.973 (15)
C11—C12	1.3984 (13)	C33—C34	1.4141 (13)
C11—H11	0.923 (15)	C33—H33	0.985 (16)
C12—C13	1.3906 (12)	C35—C40	1.4104 (12)
C12—H12	0.971 (15)	C35—C36	1.4163 (13)
C13—C14	1.5164 (12)	C36—C37	1.3691 (14)
N3—C15	1.3301 (12)	C36—H36	0.950 (16)
N3—H3A	0.873 (18)	C37—C38	1.4116 (14)
N3—H3B	0.878 (17)	C37—H37	0.987 (16)
N4—C22	1.3612 (11)	C38—C39	1.3736 (13)
N4—C21	1.3620 (12)	C38—H38	0.970 (15)
N4—H4A	0.884 (17)	C39—C40	1.4161 (12)
C15—C27	1.4349 (12)	C39—H39	0.963 (14)
C15—C16	1.4392 (13)	O1W—H1A	0.83 (2)
C16—C21	1.4121 (12)	O1W—H1B	0.861 (19)
C16—C17	1.4201 (13)	O2W—H2A	0.813 (19)
C17—C18	1.3746 (14)	O2W—H2B	0.79 (2)
C17—H17	0.934 (15)	O3W—H3C	0.79 (2)
C18—C19	1.4115 (15)	O3W—H3D	0.856 (19)
			
N1—Ni1—N2	173.93 (3)	C18—C17—H17	119.7 (9)
N1—Ni1—O1	78.42 (3)	C16—C17—H17	119.4 (9)
N2—Ni1—O1	107.10 (3)	C17—C18—C19	120.21 (9)
N1—Ni1—O4	78.42 (3)	C17—C18—H18	121.5 (9)
N2—Ni1—O4	96.15 (3)	C19—C18—H18	118.3 (9)
O1—Ni1—O4	156.71 (3)	C20—C19—C18	120.50 (9)
N1—Ni1—O5	104.16 (3)	C20—C19—H19	119.5 (10)
N2—Ni1—O5	78.28 (3)	C18—C19—H19	120.0 (10)
O1—Ni1—O5	94.00 (3)	C19—C20—C21	119.82 (9)
O4—Ni1—O5	89.26 (3)	C19—C20—H20	122.5 (9)
N1—Ni1—O8	100.69 (3)	C21—C20—H20	117.6 (9)
N2—Ni1—O8	76.88 (3)	N4—C21—C16	120.52 (8)
O1—Ni1—O8	92.00 (3)	N4—C21—C20	119.08 (8)
O4—Ni1—O8	94.68 (3)	C16—C21—C20	120.40 (8)
O5—Ni1—O8	155.12 (3)	N4—C22—C27	120.54 (8)
C1—O1—Ni1	115.38 (6)	N4—C22—C23	118.77 (8)
C7—O4—Ni1	114.59 (6)	C27—C22—C23	120.69 (8)
C8—O5—Ni1	114.08 (6)	C24—C23—C22	119.72 (8)
C14—O8—Ni1	114.13 (6)	C24—C23—H23	121.5 (9)
C2—N1—C6	122.34 (8)	C22—C23—H23	118.8 (9)
C2—N1—Ni1	118.90 (6)	C23—C24—C25	120.38 (9)
C6—N1—Ni1	118.75 (6)	C23—C24—H24	119.2 (10)
C9—N2—C13	121.37 (8)	C25—C24—H24	120.4 (10)
C9—N2—Ni1	118.02 (6)	C26—C25—C24	120.59 (9)
C13—N2—Ni1	120.08 (6)	C26—C25—H25	120.0 (9)
O2—C1—O1	126.47 (9)	C24—C25—H25	119.4 (9)
O2—C1—C2	118.90 (8)	C25—C26—C27	120.51 (8)
O1—C1—C2	114.61 (8)	C25—C26—H26	119.9 (9)
N1—C2—C3	120.31 (8)	C27—C26—H26	119.6 (9)
N1—C2—C1	112.49 (8)	C22—C27—C26	118.10 (8)
C3—C2—C1	127.18 (8)	C22—C27—C15	119.12 (8)
C2—C3—C4	118.28 (9)	C26—C27—C15	122.79 (8)
C2—C3—H3	120.8 (9)	C28—N5—H5B	119.4 (10)
C4—C3—H3	120.9 (9)	C28—N5—H5A	121.4 (11)
C5—C4—C3	120.26 (9)	H5B—N5—H5A	117.9 (15)
C5—C4—H4	119.9 (9)	C35—N6—C34	122.20 (8)
C3—C4—H4	119.8 (9)	C35—N6—H6A	118.9 (12)
C6—C5—C4	118.08 (9)	C34—N6—H6A	117.8 (12)
C6—C5—H5	119.2 (9)	N5—C28—C29	121.91 (8)
C4—C5—H5	122.7 (9)	N5—C28—C40	119.80 (8)
N1—C6—C5	120.68 (8)	C29—C28—C40	118.29 (8)
N1—C6—C7	112.46 (8)	C34—C29—C30	118.18 (8)
C5—C6—C7	126.86 (8)	C34—C29—C28	118.75 (8)
O3—C7—O4	125.73 (9)	C30—C29—C28	123.06 (8)
O3—C7—C6	118.52 (8)	C31—C30—C29	120.68 (9)
O4—C7—C6	115.76 (8)	C31—C30—H30	119.1 (9)
O6—C8—O5	127.62 (9)	C29—C30—H30	120.2 (9)
O6—C8—C9	117.00 (8)	C30—C31—C32	120.25 (9)
O5—C8—C9	115.38 (8)	C30—C31—H31	120.4 (9)
N2—C9—C10	121.18 (8)	C32—C31—H31	119.3 (9)
N2—C9—C8	113.61 (8)	C33—C32—C31	120.72 (9)
C10—C9—C8	125.21 (8)	C33—C32—H32	119.3 (9)
C9—C10—C11	118.31 (8)	C31—C32—H32	120.0 (9)
C9—C10—H10	118.8 (9)	C32—C33—C34	119.59 (9)
C11—C10—H10	122.9 (9)	C32—C33—H33	121.2 (9)
C10—C11—C12	119.83 (8)	C34—C33—H33	119.2 (9)
C10—C11—H11	120.2 (9)	N6—C34—C33	118.67 (8)
C12—C11—H11	120.0 (9)	N6—C34—C29	120.76 (8)
C13—C12—C11	118.26 (8)	C33—C34—C29	120.56 (9)
C13—C12—H12	121.7 (9)	N6—C35—C40	120.56 (8)
C11—C12—H12	120.0 (9)	N6—C35—C36	118.90 (8)
N2—C13—C12	121.04 (8)	C40—C35—C36	120.52 (8)
N2—C13—C14	112.51 (7)	C37—C36—C35	119.41 (9)
C12—C13—C14	126.41 (8)	C37—C36—H36	121.7 (10)
O7—C14—O8	125.99 (9)	C35—C36—H36	118.9 (10)
O7—C14—C13	118.26 (8)	C36—C37—C38	120.74 (9)
O8—C14—C13	115.75 (8)	C36—C37—H37	120.9 (9)
C15—N3—H3A	121.6 (12)	C38—C37—H37	118.3 (9)
C15—N3—H3B	121.1 (11)	C39—C38—C37	120.27 (9)
H3A—N3—H3B	117.2 (15)	C39—C38—H38	119.4 (9)
C22—N4—C21	122.33 (8)	C37—C38—H38	120.3 (9)
C22—N4—H4A	117.4 (11)	C38—C39—C40	120.57 (9)
C21—N4—H4A	120.0 (11)	C38—C39—H39	118.2 (8)
N3—C15—C27	120.41 (8)	C40—C39—H39	121.1 (8)
N3—C15—C16	121.15 (8)	C35—C40—C39	118.39 (8)
C27—C15—C16	118.44 (8)	C35—C40—C28	119.18 (8)
C21—C16—C17	118.21 (8)	C39—C40—C28	122.30 (8)
C21—C16—C15	119.03 (8)	H1A—O1W—H1B	104.2 (17)
C17—C16—C15	122.73 (8)	H2A—O2W—H2B	105.1 (18)
C18—C17—C16	120.87 (9)	H3C—O3W—H3D	108.3 (17)
			
N1—Ni1—O1—C1	−3.81 (7)	C9—N2—C13—C14	178.01 (8)
N2—Ni1—O1—C1	173.57 (7)	Ni1—N2—C13—C14	6.49 (10)
O4—Ni1—O1—C1	−10.03 (12)	C11—C12—C13—N2	0.10 (13)
O5—Ni1—O1—C1	−107.47 (7)	C11—C12—C13—C14	−177.60 (8)
O8—Ni1—O1—C1	96.69 (7)	Ni1—O8—C14—O7	−172.58 (7)
N1—Ni1—O4—C7	−1.21 (7)	Ni1—O8—C14—C13	7.53 (10)
N2—Ni1—O4—C7	−178.46 (7)	N2—C13—C14—O7	170.84 (8)
O1—Ni1—O4—C7	5.00 (12)	C12—C13—C14—O7	−11.29 (14)
O5—Ni1—O4—C7	103.41 (7)	N2—C13—C14—O8	−9.27 (11)
O8—Ni1—O4—C7	−101.19 (7)	C12—C13—C14—O8	168.60 (9)
N1—Ni1—O5—C8	170.05 (6)	N3—C15—C16—C21	−177.27 (9)
N2—Ni1—O5—C8	−4.26 (6)	C27—C15—C16—C21	2.02 (13)
O1—Ni1—O5—C8	−110.90 (6)	N3—C15—C16—C17	0.48 (14)
O4—Ni1—O5—C8	92.18 (6)	C27—C15—C16—C17	179.76 (9)
O8—Ni1—O5—C8	−7.38 (11)	C21—C16—C17—C18	0.23 (14)
N1—Ni1—O8—C14	−177.64 (6)	C15—C16—C17—C18	−177.54 (9)
N2—Ni1—O8—C14	−3.33 (6)	C16—C17—C18—C19	−0.57 (16)
O1—Ni1—O8—C14	103.77 (7)	C17—C18—C19—C20	0.39 (16)
O4—Ni1—O8—C14	−98.56 (7)	C18—C19—C20—C21	0.13 (15)
O5—Ni1—O8—C14	−0.19 (11)	C22—N4—C21—C16	0.55 (13)
O1—Ni1—N1—C2	3.91 (7)	C22—N4—C21—C20	−179.16 (8)
O4—Ni1—N1—C2	−178.59 (7)	C17—C16—C21—N4	−179.41 (8)
O5—Ni1—N1—C2	95.16 (7)	C15—C16—C21—N4	−1.56 (13)
O8—Ni1—N1—C2	−85.94 (7)	C17—C16—C21—C20	0.30 (13)
O1—Ni1—N1—C6	−177.21 (7)	C15—C16—C21—C20	178.15 (8)
O4—Ni1—N1—C6	0.29 (7)	C19—C20—C21—N4	179.24 (9)
O5—Ni1—N1—C6	−85.96 (7)	C19—C20—C21—C16	−0.48 (14)
O8—Ni1—N1—C6	92.93 (7)	C21—N4—C22—C27	−0.01 (13)
O1—Ni1—N2—C9	97.94 (7)	C21—N4—C22—C23	−179.65 (8)
O4—Ni1—N2—C9	−80.63 (7)	N4—C22—C23—C24	−179.61 (8)
O5—Ni1—N2—C9	7.33 (6)	C27—C22—C23—C24	0.74 (13)
O8—Ni1—N2—C9	−174.02 (7)	C22—C23—C24—C25	−0.06 (14)
O1—Ni1—N2—C13	−90.26 (7)	C23—C24—C25—C26	−0.96 (14)
O4—Ni1—N2—C13	91.17 (7)	C24—C25—C26—C27	1.28 (14)
O5—Ni1—N2—C13	179.13 (7)	N4—C22—C27—C26	179.94 (8)
O8—Ni1—N2—C13	−2.22 (6)	C23—C22—C27—C26	−0.42 (13)
Ni1—O1—C1—O2	−178.27 (8)	N4—C22—C27—C15	0.51 (13)
Ni1—O1—C1—C2	3.09 (10)	C23—C22—C27—C15	−179.85 (8)
C6—N1—C2—C3	−0.99 (14)	C25—C26—C27—C22	−0.60 (13)
Ni1—N1—C2—C3	177.85 (7)	C25—C26—C27—C15	178.82 (9)
C6—N1—C2—C1	177.73 (8)	N3—C15—C27—C22	177.79 (8)
Ni1—N1—C2—C1	−3.44 (10)	C16—C15—C27—C22	−1.50 (12)
O2—C1—C2—N1	−178.73 (9)	N3—C15—C27—C26	−1.62 (14)
O1—C1—C2—N1	0.02 (12)	C16—C15—C27—C26	179.09 (8)
O2—C1—C2—C3	−0.13 (15)	N5—C28—C29—C34	176.98 (8)
O1—C1—C2—C3	178.62 (9)	C40—C28—C29—C34	−4.17 (12)
N1—C2—C3—C4	1.59 (15)	N5—C28—C29—C30	−2.62 (14)
C1—C2—C3—C4	−176.92 (9)	C40—C28—C29—C30	176.22 (8)
C2—C3—C4—C5	−0.51 (15)	C34—C29—C30—C31	1.09 (13)
C3—C4—C5—C6	−1.13 (15)	C28—C29—C30—C31	−179.30 (9)
C2—N1—C6—C5	−0.74 (14)	C29—C30—C31—C32	−0.67 (14)
Ni1—N1—C6—C5	−179.58 (7)	C30—C31—C32—C33	−0.61 (14)
C2—N1—C6—C7	179.35 (8)	C31—C32—C33—C34	1.41 (14)
Ni1—N1—C6—C7	0.52 (10)	C35—N6—C34—C33	−176.15 (8)
C4—C5—C6—N1	1.78 (14)	C35—N6—C34—C29	4.00 (13)
C4—C5—C6—C7	−178.33 (9)	C32—C33—C34—N6	179.19 (8)
Ni1—O4—C7—O3	−178.14 (8)	C32—C33—C34—C29	−0.96 (13)
Ni1—O4—C7—C6	1.82 (10)	C30—C29—C34—N6	179.57 (8)
N1—C6—C7—O3	178.38 (8)	C28—C29—C34—N6	−0.06 (13)
C5—C6—C7—O3	−1.52 (14)	C30—C29—C34—C33	−0.28 (13)
N1—C6—C7—O4	−1.58 (12)	C28—C29—C34—C33	−179.91 (8)
C5—C6—C7—O4	178.52 (9)	C34—N6—C35—C40	−3.45 (13)
Ni1—O5—C8—O6	179.89 (8)	C34—N6—C35—C36	175.03 (8)
Ni1—O5—C8—C9	0.92 (10)	N6—C35—C36—C37	−176.16 (9)
C13—N2—C9—C10	0.07 (13)	C40—C35—C36—C37	2.32 (14)
Ni1—N2—C9—C10	171.76 (7)	C35—C36—C37—C38	0.56 (15)
C13—N2—C9—C8	179.37 (8)	C36—C37—C38—C39	−2.15 (15)
Ni1—N2—C9—C8	−8.94 (10)	C37—C38—C39—C40	0.84 (15)
O6—C8—C9—N2	−174.13 (8)	N6—C35—C40—C39	174.90 (8)
O5—C8—C9—N2	4.96 (12)	C36—C35—C40—C39	−3.55 (13)
O6—C8—C9—C10	5.13 (14)	N6—C35—C40—C28	−1.02 (13)
O5—C8—C9—C10	−175.78 (9)	C36—C35—C40—C28	−179.47 (8)
N2—C9—C10—C11	−0.28 (13)	C38—C39—C40—C35	1.97 (13)
C8—C9—C10—C11	−179.49 (8)	C38—C39—C40—C28	177.75 (9)
C9—C10—C11—C12	0.39 (14)	N5—C28—C40—C35	−176.42 (8)
C10—C11—C12—C13	−0.31 (13)	C29—C28—C40—C35	4.71 (12)
C9—N2—C13—C12	0.02 (13)	N5—C28—C40—C39	7.83 (13)
Ni1—N2—C13—C12	−171.51 (7)	C29—C28—C40—C39	−171.04 (8)

### Hydrogen-bond geometry (Å, °)

**Table:**

*D*—H···*A*	*D*—H	H···*A*	*D*···*A*	*D*—H···*A*
N3—H3A···O2W^i^	0.873 (18)	2.053 (18)	2.8793 (12)	157.6 (16)
N3—H3B···O7^ii^	0.878 (17)	2.109 (17)	2.9337 (11)	156.1 (15)
N4—H4A···O6	0.884 (17)	1.838 (17)	2.7214 (11)	178.1 (16)
N5—H5A···O3^iii^	0.894 (17)	1.925 (17)	2.7945 (11)	163.9 (16)
N5—H5B···O5	0.889 (17)	2.046 (17)	2.9096 (11)	163.6 (15)
N6—H6A···O3W	0.836 (18)	1.863 (18)	2.6903 (11)	170.3 (18)
O1W—H1A···O7	0.83 (2)	1.99 (2)	2.8138 (11)	171 (2)
O1W—H1B···O7^iv^	0.861 (19)	1.955 (19)	2.8161 (11)	178.7 (17)
O2W—H2A···O8^v^	0.813 (19)	2.067 (19)	2.8717 (11)	170.1 (17)
O2W—H2B···O2	0.79 (2)	2.02 (2)	2.8033 (11)	177 (2)
O3W—H3C···O3^vi^	0.79 (2)	1.97 (2)	2.7595 (11)	169.8 (19)
O3W—H3D···O1W^vii^	0.856 (19)	1.879 (19)	2.7328 (11)	175.4 (17)
C30—H30···O3^iii^	0.931 (16)	2.437 (16)	3.3386 (12)	163 (14)

Symmetry codes: (i) −*x*+1, −*y*, −*z*+1; (ii) *x*+1, *y*, *z*+1; (iii) −*x*+1, −*y*, −*z*; (iv) −*x*, −*y*+1, −*z*; (v) −*x*, −*y*, −*z*; (vi) *x*, *y*, *z*+1; (vii) −*x*+1, −*y*+1, −*z*+1.
